# Monocyte-derived tissue transglutaminase in multiple sclerosis patients: reflecting an anti-inflammatory status and function of the cells?

**DOI:** 10.1186/s12974-017-1035-y

**Published:** 2017-12-21

**Authors:** Claudia Sestito, John J. P. Brevé, Marja C. J. A. van Eggermond, Joep Killestein, Charlotte E. Teunissen, Joram van Rossum, Micha M. M. Wilhelmus, Benjamin Drukarch, Peter J. van den Elsen, Anne-Marie van Dam

**Affiliations:** 1 Department of Anatomy and Neurosciences, Amsterdam Neuroscience, VU University Medical Center, Postbus 7057, 1007 MB Amsterdam, the Netherlands; 2 Department of Immunohematology and Blood Transfusion, Leiden University Medical Center, Leiden, the Netherlands; 3 Department of Neurology, VU University Medical Center, Amsterdam, the Netherlands; 4 Department of Clinical Chemistry, VU University Medical Center, Amsterdam, the Netherlands; 5 Department of Pathology, VU University Medical Center, Amsterdam, the Netherlands; 6 Present Address: Brain Plasticity Group, Center for Neuroscience, Swammerdam Institute for Life Sciences, University of Amsterdam, Science Park 904, Amsterdam, the Netherlands

**Keywords:** Multiple sclerosis, Tissue transglutaminase, Inflammation, Human monocytes, Cytokine, Adhesion/migration

## Abstract

**Background:**

Leukocyte infiltration into the central nervous system is an important feature of multiple sclerosis (MS) pathology. Among the infiltrating cells, monocytes comprise the largest population and are considered to play a dual role in the course of the disease. The enzyme tissue transglutaminase (TG2), produced by monocytes, plays a central role in monocyte adhesion/migration in animal models of MS. In the present study, we questioned whether TG2 expression is altered in monocytes from MS patients compared to healthy control (HC) subjects. Moreover, we determined the inflammatory status of these TG2-expressing monocytes, what inflammatory factor regulates TG2 expression, and whether TG2 can functionally contribute to their adhesion/migration processes.

**Methods:**

Primary human monocytes from MS patients and HC subjects were collected, RNA isolated and subjected to qPCR analysis. Human THP-1 monocytes were lentivirally transduced with TG2 siRNA or control and treated with various cytokines. Subsequently, mRNA levels of inflammatory factors, adhesion properties, and activity of RhoA were analyzed in interleukin (IL)-4-treated monocytes.

**Results:**

TG2 mRNA levels are significantly increased in monocytes derived from MS patients compared to HC subjects. In addition, correlation analyses indicated that TG2-expressing cells display a more anti-inflammatory, migratory profile in MS patients. Using THP-1 monocytes, we observed that IL-4 is a major trigger of TG2 expression in these cells. Furthermore, knockdown of TG2 expression leads to a pro-inflammatory profile and reduced adhesion/migration properties of IL-4-treated monocytes.

**Conclusions:**

TG2-expressing monocytes in MS patients have a more anti-inflammatory profile. Furthermore, TG2 mediates IL-4-induced anti-inflammatory status in THP-1 monocytes, adhesion, and cytoskeletal rearrangement in vitro. We thus propose that IL-4 upregulates TG2 expression in monocytes of MS patients, driving them into an anti-inflammatory status.

**Electronic supplementary material:**

The online version of this article (10.1186/s12974-017-1035-y) contains supplementary material, which is available to authorized users.

## Background

Multiple sclerosis (MS) is a chronic neurological disorder affecting mostly young adults that leads to, e.g., sensory, motor, and cognitive deficits [1]. Pathologically, it is characterized by inflammation, demyelination, and axonal loss in the central nervous system (CNS) [2]. Although the pathophysiology is not well understood, MS results in leukocyte infiltration into the CNS parenchyma which requires passage of the blood-brain barrier (BBB) [3]. This process is initiated by the secretion of cytokines and chemokines which induce leukocyte activation by stimulating conformational changes in the integrins, key regulators of the adhesion/migration cascade. These changes, together with cytoskeleton rearrangement and the secretion of matrix metalloproteinase (MMP) capable of cleaving components of the extracellular matrix (ECM), allow the arrest and finally the migration of the cells into the CNS [4, 5]. Monocytes and monocyte-derived macrophages constitute the major cell type in the perivascular infiltrates characteristic of several neuroinflammatory diseases including MS [6–9] and are considered to play a pivotal role in MS pathology. Indeed, depletion of monocytes and macrophages or inhibition of monocyte recruitment to the CNS leads to a reduction of clinical symptoms and pathology in animals suffering from experimental autoimmune encephalomyelitis (EAE), an animal model of MS [7, 10, 11]. Once migrated into the CNS, the recruited monocytes/macrophages, together with the locally activated microglia, release proteases, pro-inflammatory cytokines, and reactive oxygen species, contributing to myelin damage and, ultimately, axonal damage [12, 13]. Although monocytes/macrophages are thought to primarily have detrimental effects on MS pathogenesis and development, other studies point toward a dual role for these cells since it has been proven that they can also contribute to the clearance of tissue debris and promote tissue repair by secreting growth factors and anti-inflammatory cytokines, such as nerve growth factor (NGF) and interleukin (IL)-10 [14–16]. Furthermore, administration of anti-inflammatory-tuned monocytes to animals suffering from severe EAE ameliorates their clinical disease status [17–19].

Tissue transglutaminase, or transglutaminase 2 (TG2), is a calcium-dependent protein-crosslinking enzyme whose expression and activity can be increased by inflammatory mediators in various cell types, including monocytes [20–23]. A recent study from our group demonstrated TG2 immunoreactivity in human active MS lesions in infiltrating cells of which some express MHC-II. In addition, we observed in an animal experimental MS model that TG2 is present in infiltrating monocytes and contributes to pathology by promoting monocyte migration into the CNS [24]. Of interest is that TG2 has also been shown to be involved in the adhesion of monocytes onto ECM proteins in vitro (i.e., fibronectin, FN) as it serves as a β-integrin-associated co-receptor promoting binding to fibronectin [25].

Thus, we hypothesize that in monocytes of MS patients, TG2 expression is altered due to the inflammatory environment. Consequently, TG2 expressed by monocytes could contribute to adhesion and migration processes that are essential for cells entering the CNS, resulting in MS pathology. In the present study, we studied TG2 expression in monocytes from MS patients and healthy control (HC) subjects and we determined the inflammatory status of the TG2-expressing monocytes. In addition, using the human THP-1 monocyte cell line, we studied the regulation of TG2 expression by inflammatory mediators and its possible functional implication in monocyte adhesion/migration processes.

## Methods

### Isolation of primary human monocytes

Peripheral blood mononuclear cells (PBMCs) were obtained from 15 MS patients (13 relapsing-remitting and 3 primary progressive, RR-MS, and PP-MS respectively) diagnosed according to the Poser or McDonald criteria [26] and 10 HC subjects (Table 1). MS patients were recruited from the VU University Medical Center (VUmc) outpatient clinic while participating in different prospective cohort studies. Age and sex-matched control subjects were recruited from the Leiden University Medical Center (LUMC). All participants gave informed consent, and the study was approved by the Medical Research Ethics Committees of the VUmc and LUMC, respectively. At time of blood collection, the activity status of the disease was not measured. Peripheral blood was drawn by venipuncture and collected into sodium citrate tubes (Greiner, Kremsmünster, Austria). One part of blood was diluted with one part of buffer consisting of phosphate-buffered saline (PBS) + 10% *v*/*v* GPO (Sanquin, Amsterdam, The Netherlands) + 10% *v*/*v* sodium citrate. PBMCs were isolated by density centrifugation using Ficoll Isopaque PLUS (GE Healthcare Biosciences, Uppsala, Sweden). Monocytes were subsequently isolated from the PBMCs by anti-CD14 magnetic beads according to the manufacturer’s instruction (MACS; Milteny Biotech, Bergisch Gladbach, Germany).

**Table 1:** Patient information

Subjects	Number	Female/male	Age (years) ± SD	Type MS	DMT
HC	Total 10	4/6	40 ± 12		
	1	Female	25	–	–
	2	Female	49	–	–
	3	Female	25	–	–
	4	Female	46	–	–
	5	Male	50	–	–
	6	Male	42	–	–
	7	Male	30	–	–
	8	Male	39	–	–
	9	Male	33	–	–
	10	Male	60	–	–
MS	Total 15	8/7	45 ± 16		
	11	Female	73	PP-MS	Untreated
	12	Male	51	PP-MS	Untreated
	13	Male	56	PP-MS	Untreated
	14	Female	25	RR-MS	Interferon beta-1a
	15	Female	71	RR-MS	Untreated
	16	Female	24	RR-MS	Glatiramer acetate
	17	Female	49	RR-MS	Glatiramer acetate
	18	Male	30	RR-MS	Interferon beta-1a
	19	Male	48	RR-MS	Natalizumab
	20	Female	30	RR-MS	Untreated
	21	Female	32	RR-MS	Untreated
	22	Female	46	RR-MS	Untreated
	23	Male	45	RR-MS	Untreated
	24	Male	58	RR-MS	Untreated
	25	Male	32	RR-MS	Untreated

*HC* healthy controls, *MS* multiple sclerosis, *PP-MS* primary progressive MS, *RR-MS* relapsing-remitting MS, *SD* standard deviation, *DMT* disease-modifying therapies

### Cell culture and treatment

THP-1 cells (human monocytic cell line) were maintained in complete RPMI 1640 medium (Gibco, Waltham, Massachusetts, USA) containing 10% heat-inactivated fetal calf serum (PAA), penicillin (50 units/ml, Gibco), streptomycin (50 μg/ml, Gibco), and L-glutamine (2 mM, Gibco) at 37 °C in humidified air containing 5% CO_2_.

Scramble control (SCR) and TG2-knockdown (TG2-KD) THP-1 cell lines were generated by double, subsequent lentiviral transduction (MOI 1) with either a scramble or hTG2 specific short hairpin RNA (shRNA) (scramble: sc-108080, hTG2: sc-37514-V, Santa Cruz, Dallas, Texas, USA) for 24 h for each infection.

To create stable knockdown cell lines, cells were selected with 2 mg/ml puromycin (Sigma-Aldrich, Saint Louis, Missouri, USA). Subsequently, the cells were characterized by western blot analysis and semi-quantitative real-time PCR (qPCR) (see below) and used for functional assays. Stable cell lines were maintained in complete RPMI in presence of 2 mg/ml puromycin.

THP-1 cells were either untreated or treated with 50 ng/ml of the human recombinant IL-4, IL-10, IL-1β, or tumor necrosis factor (TNF)-α (BioLegend, San Diego, USA) for 24 h in culture medium. The dose of cytokine used was based on previous studies from our group [24, 27]. The incubation period was chosen based on the time-dependent TG2 expression in THP-1 cells showing that a 24-h treatment with cytokines resulted in the most increased TG2 expression (data not shown).

### RNA isolation and cDNA synthesis from primary human monocytes

Primary human monocytes were centrifuged for 5 min at 500×*g*. Cells were lysed in RNAbee (Tel-Test, Friendswood, USA) according to the manufacturer’s instructions. Lysates were stored at − 80 °C until further processing. Total RNA was extracted according to the manufacturer’s instructions. The RNA concentration was determined by measuring the absorbance at 260 nm (NanoDrop ND-100 spectophotometer; Thermo Fisher scientific, Waltham, USA) and when approved, samples were included for cDNA synthesis. From 1 μg total RNA, cDNA was synthesized using Superscript III (Invitrogen, California, USA) with 250 ng random hexamers (Promega, Wisconsin, USA) and according to the manufacturer’s instructions.

### RNA isolation and cDNA synthesis from THP-1 cells

THP-1 cells were homogenized in Trizol reagent (Invitrogen) and total RNA was isolated as described by the manufacturer. RNA concentration was determined by measuring the absorbance at 260 nm (NanoDrop ND-1000 spectrophotometer) and when approved, samples were included for cDNA synthesis. Then, 1 μg of total RNA was reverse-transcribed into cDNA using the High-Capacity cDNA Reverse Transcription kit (Life Technologies, California, USA), using 0.5 μg oligo-dT primers and according to the manufacturer’s instructions.

### Semi-quantitative real-time PCR (qPCR)

For qPCR, the Power SYBR Green Master Mix (Life Technologies) was used. Primers were purchased from Eurogentec (Liège, Belgium) and qPCR was performed in MicroAmp Optical 96-well Reaction Plates (Applied Biosystems, California, USA) on a StepOnePlus Real-Time PCR system (Applied Biosystems). The reaction mixture (20 μl) was composed of 1× Power SYBR Green buffer (Applied Biosystems), 3.75 pmol of each primer (Table 2), and 100 ng cDNA. The thermal cycling conditions were an initial 10 min at 95 °C followed by 40 cycles of 15 s at 95 °C and 1 min at 60 °C. The specificity of the reaction was checked by melt curve analysis of the individual qPCR reaction. The relative expression level of the target genes was determined by the LinRegPCR software (http://www.hfrc.nl, downloads, applications, lin reg PCR, version 2017 ) using the following calculation *N*0 = Nq/*E*Cq (*N*0 = target quantity, Nq = fluorescence threshold value, *E* = mean PCR efficiency per amplicon, Cq = threshold cycle) [28], after which the value was normalized relative to the geometric mean of the mRNA levels of glyceraldehyde-3-phosphate-dehydrogenase (GAPDH) and polymerase (RNA) II polypeptide F (POLR2F). We chose GAPDH and POLR2F as reference genes based on the results of the GeNorm software analysis (version 3.5) in which the stability of six different human housekeeping genes (GAPDH, MRIP, POLR2F, HPRT1, PGK1, SDHA) was assessed in our primary human monocytes. Regarding the THP-1 cells, the value was normalized relative to the mRNA levels of GAPDH as previously described [29].

**Table 2:** Primer sequences

Gene	Forward	Reverse
TG2	5’AGAGGAGCGGCAGGAGTATG 3’	5’AGGATCCCATCTTCAAACTGC 3’
GAPDH	5’TCAAGGGCATCCTGGGCTAC 3’	5’CGTCAAAGGTGGAGGAGTGG 3’
POLR2F	5’GAACTCAAGGCCCGAAAG 3’	5’TGATGATGAGCTCGTCCAC 3’
IL-1β	5’TACAGCTGGAGAGTGTAGATC 3’	5’CAAATTCCAGCTTGTTATTG 3’
TNF-α	5’CCCAGGCAGTCAGATCATCTTC 3’	5’CTCTCAGCTCCACGCCATTG 3’
IL-1ra	5’TCATCCGCTCAGACAGTGGC 3’	5’AGCTTCCATCGCTGTGCAGA 3’
TGF-β1	5’CTTTCCTGCTTCTCATGGCC 3’	5’CCGTGGAGCTGAAGCAATAG 3’
β1 integrin	5’TGTGGAGGAAATGGTGTTTGC 3’	5’TCTGTCCGTTGCTGGCTTCA 3’
β3 integrin	5’CAATGCCACCTGCCTCAACA 3’	5’GAGTCTTCATAGTACTGGAATC 3’
MMP-2	5’AAGGCCAAGTGGTCCGTGTG 3’	5’GTGCAGCTGTTGTACTCCTTGC 3’

*TG2* Transglutaminase 2, *GAPDH* glyceraldehyde-3-phosphate-dehydrogenase, *POLR2F* polymerase (RNA) II polypeptide F, *IL-1β* interleukin-1β, *TNF-α* tumor necrosis factor-α, *IL-1ra* interleukin-1 receptor antagonist, *TGF-β* transforming growth factor-β1, *MMP-2* matrix metalloproteinase s2

### Western blotting (WB)

To determine TG2 protein expression, cells were homogenized in ice-cold RIPA buffer containing 150 mM NaCl, 20 mM Tris-HCl (pH 7.4), 1% NP-40, 1% sodium deoxycholate, 1 mM EDTA, 0.1% sodium dodecyl sulfate (SDS), 15 μM Pepstatin A, 100 μM phenylmethylsulfonyl fluoride (PMSF), 0.3 μM aprotinin, and 15 μM leupeptin. Cell lysates were cleared by centrifugation (14.000 rpm for 10 min at 4 °C). Protein concentration of the supernatant was determined by the bicinchoninic acid (BCA) method according to the instructions of the manufacturer (Pierce Biotechnology, Waltham, Massachusetts, USA).

Fifty micrograms of protein were denaturated by adding Laemmli buffer (0.012% bromophenol blue, 5% glycerol, 1.6% SDS, 125 mM Tris HCl pH 6.8) and 50 mM dithiothreitol (DTT). After boiling, the mixture was loaded on a 10% SDS-polyacrylamide gel and transferred onto a nitrocellulose membrane (Li-cor Biosciences, Lincoln, Nebraska, USA). Membranes were incubated overnight with the following primary antibodies: mouse anti-β-actin (1:10,000; Abcam, Cambridge, UK) and mouse anti-TG2 (Ab3, 1:2000; Thermo Scientific, Waltham, Massachusetts, USA) diluted in Li-cor buffer. For subsequent antigen detection, the membranes were incubated for 1 h at room temperature (RT) with corresponding IgG’s labeled with IRDye 800CW or IRDye 680LT (1:10,000; Li-cor buffer) and subsequently scanned to detect fluorescence emission at 800 or 680 nm, respectively, using an Odyssey infrared imaging system (Li-Cor Biosciences). The signal intensity was measured using the Odyssey Sa Infrared scanning software (version 1.1, Li-Cor Biosciences).

### Adhesion assay

SCR or TG2-KD THP-1 monocytes were treated with 50 ng/ml IL-4. After 24 h treatment, cells were centrifuged and resuspended in adhesion medium (RPMI 1640 medium + 0.5% bovine serum albumin +25 mM HEPES). 5 × 10^4^ of the treated cells were then added to a 96-well plate coated with 2 μg/cm^2^ bovine FN (Sigma-Aldrich). Cells were allowed to adhere to the FN layer at 37° on an orbital shaker (Vibramax 100, Heidolph, Germany) at 300 rpm. After 1 h, the non-adherent cells were removed and the adherent cells were labeled with 0.5 μM calcein (Invitrogen) in the adhesion medium for 10 min at 37°. Then, excess of calcein was removed and cells were washed four times with PBS and lysed in 0.1 M NaOH. The fluorescence (emission 485 nm; extinction 520 nm) was then measured using a spectrophotometer (Fluostar Galaxy Microplate Reader, BMG Lab Technologies, Germany) and using FluoStar Software (BMG Lab Technologies).

### RhoA GTPase activity assay

To determine the effect of TG2 on cytoskeletal reorganization, we measured RhoA GTPase activity. 2 × 10^6^ SCR or TG2-KD THP-1 monocytes were treated with 50 ng/ml IL-4 for 24 h. Thereafter, cells were lysed with lysis buffer (50 mM Tris, pH 7.6, 150 mM NaCl, 1% Triton X-100, 20 mM MgCl2, 5 μg/ml Pepstatin A, 100 μM PMSF, 5 μg/ml aprotinin, and 5 μg/ml leupeptin). Of the cleared lysates, 30 μg was stored to determine the total amount of RhoA (total cell lysate). Remaining protein lysates were incubated with 60 μg bacterially produced GST-RBD (Rho Binding Domain of Rhotekin) bound to glutathione-agarose beads (Cytoskeleton, USA) for 30 min at 4°. Beads were washed four times with lysis buffer containing 50 mM Tris, pH 7.6, 150 mM NaCl, 1% Triton X-100, 10 mM MgCl_2_. Bound proteins were eluted in 30 μl SDS Laemmli buffer and analyzed in parallel with the total cell lysate on a 12.5% SDS PAGE. The active form of RhoA and total RhoA were detected with a mouse monoclonal antibody (1:250, Santa Cruz Biotechnology) and were visualized as described under western blotting.

### Statistical analysis

In the HC or MS patient-derived monocytes, the sample size varied between gene expression measurements due to availability of mRNA or cDNA or based on unreliable qPCR outcome (e.g., extreme values). Normal distribution of the data was tested using the Shapiro-Wilk procedure. When data from MS patients and HC were not normally distributed, either the logarithmic (for IL-1β and TNF-α) or the cubic root transformed parameter (for β3 Integrin) was used in order to normalize the variables. Subsequently, normally distributed data were analyzed by independent Student *t* test. Correlation analysis was assessed using Pearson correlation analysis. The data obtained with the THP-1 cells all met the criteria of normal distribution. Subsequently, one-way analysis of variance (ANOVA) followed by Bonferroni post hoc test was performed for multiple comparisons while for two group analysis, an independent Student *t* test was performed. Results were considered to be statistically significant if *p* < 0.05. All statistical analyses were performed using SPSS software, version 20.0 (IBM Corp, NY, USA).

## Results

### TG2 mRNA levels were increased in MS patient-derived monocytes compared to HC subject-derived monocytes

**Fig. 1:**
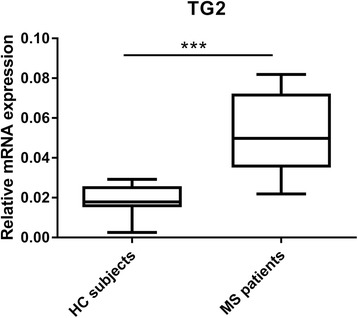
TG2 expression in MS patient/HC subject-derived monocytes. qPCR analysis was performed to detect TG2 (HC *N* = 8/MS *N* = 15) mRNA levels in primary human monocytes isolated from MS patients and HC subjects. Data are shown in box-and-whiskers plots in which the median is represented by a horizontal line within the box and the lower and upper whiskers represent the 5 and 95 percentiles. ***p < 0.001. MS multiple sclerosis, HC healthy controls

At first, we established that there was no significant difference in TG2 expression between untreated and drug-treated MS patients (see Additional file 1). Therefore, considering the relative low number of MS patients, all MS patient samples were further assessed as one group. When comparing TG2 mRNA levels between HC subjects and MS patients, there was a significant increase in TG2 expression in MS patient-derived monocytes compared to HC subject-derived monocytes (*p* < 0.001) (Fig. 1).

### MS patient-derived monocytes showed a decrease in IL-1β mRNA and an increase in β3 integrin mRNA levels compared to HC subject-derived monocytes

**Fig. 2:**
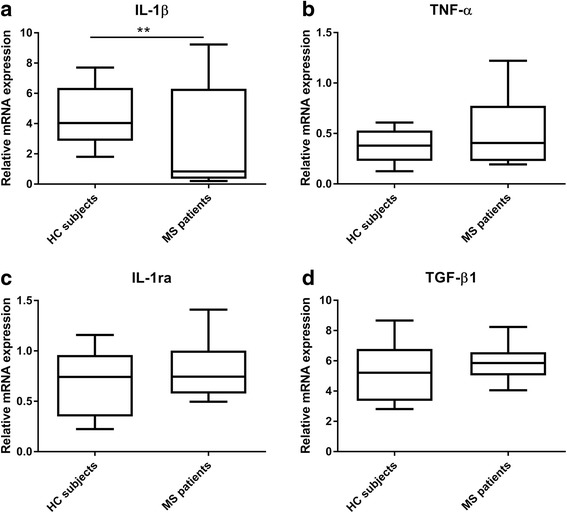
IL-1β, TNF-α, IL-1ra, and TGF-β1 mRNA levels in MS patient/HC subject-derived monocytes. qPCR analysis was performed to detect **a** IL-1β (HC *N* = 9/MS *N* = 15), **b** TNF-α (HC *N* = 10/MS *N* = 13), **c** IL-1ra (HC *N* = 8/MS *N* = 14), and **d** TGF-β1 (HC *N* = 8/MS *N* = 14) mRNA levels in primary monocytes from MS patients and HC subjects. Data are shown in box-and-whiskers plots in which the median is represented by a horizontal line within the box, and the lower and upper whiskers represent the 5 and 95 percentiles. **p < 0.01. MS multiple sclerosis, HC healthy controls

**Fig. 3:**
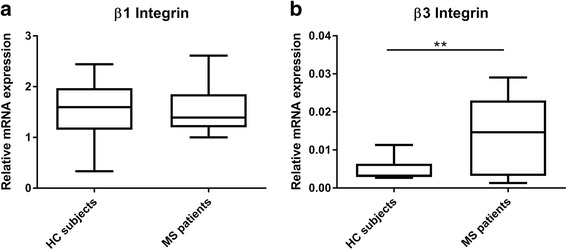
β1 and β3 integrin mRNA levels in MS patient/HC subject-derived monocytes. qPCR analysis was performed to detect **a** β1 integrin (HC *N* = 8/MS *N* = 14) and **b** β3 integrin (HC *N* = 7/MS *N* = 15) mRNA levels in primary monocytes from MS patients and HC subjects. Data are shown in box-and-whiskers plots in which the median is represented by a horizontal line within the box, and the lower and upper whiskers represent the 5 and 95 percentiles. **p < 0.01. MS multiple sclerosis, HC healthy controls

Subsequently, we aimed to study the inflammatory phenotype of MS patient/HC subject-derived monocytes. We measured the expression of several pro- and anti-inflammatory mediators. Interestingly, MS patient-derived monocytes displayed a significant decrease in pro-inflammatory IL-1β expression compared to HC subject-derived monocytes (*p* = 0.003) (Fig. 2a), whereas pro-inflammatory TNF-α and anti-inflammatory IL-1 receptor antagonist (IL-1ra) and transforming growth factor (TGF)-β1 mRNA levels were not significantly different (Fig. 2b–d). In addition, we examined whether the expression of two relevant members of the β integrin family, β1 and β3, were also affected in monocytes derived from MS patients versus HC subjects. While β1 integrin mRNA levels were not significantly different between the two groups (Fig. 3a), we observed a significant increase in β3 integrin mRNA levels in MS patient-derived monocytes compared to HC subject-derived monocytes (*p* = 0.01) (Fig. 3b).

### TG2 mRNA levels correlated with those of inflammatory mediators in MS patient-derived monocytes

**Fig. 4:**
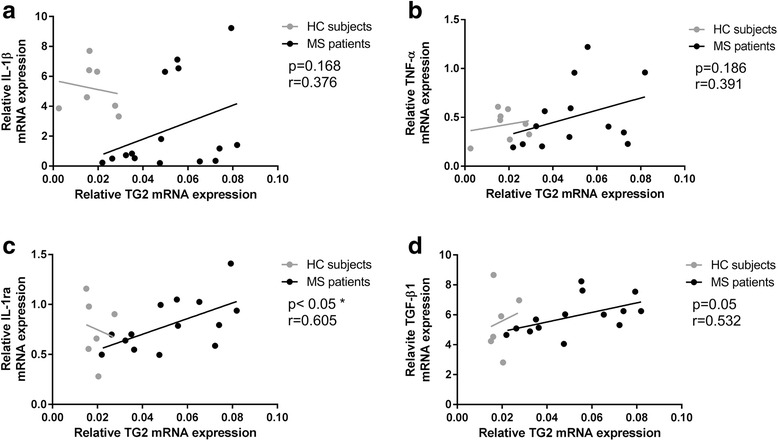
Correlation of TG2 mRNA levels with pro/anti-inflammatory mediators. Pearson correlation analysis was performed to correlate TG2 mRNA levels with **a** IL-1β, **b** TNF-α, **c** IL-1ra, and **d** TGF-β1mRNA levels in MS patient-derived monocytes (*black line*) and HC subject-derived monocytes (*gray line*). *p < 0.05. MS multiple sclerosis, HC healthy controls

Next, to determine the phenotype of TG2-expressing monocytes derived from MS patients and HC subjects, we correlated TG2 mRNA levels with mRNA levels of pro- and anti-inflammatory mediators. In MS patient-derived monocytes (Fig. 4 black line), no significant correlation was observed between TG2 mRNA levels and either IL-1β mRNA levels (*r* = 0.376, *p* = 0.168) or TNFα mRNA levels (*r* = 0.391, *p* = 0.186) (Fig. 4a, b). However, TG2 mRNA levels correlated positively with the anti-inflammatory mediators IL-1ra (*r* = 0.605, *p* = 0.02) and TGF-β1 (*r* = 0.532, *p* = 0.05) (Fig. 4c, d). None of these correlations was observed in HC subject-derived monocytes (Fig. 4a–d, gray line).

### TG2 expression was upregulated in IL-4-treated monocytes and sustained an anti-inflammatory phenotype

**Fig. 5:**
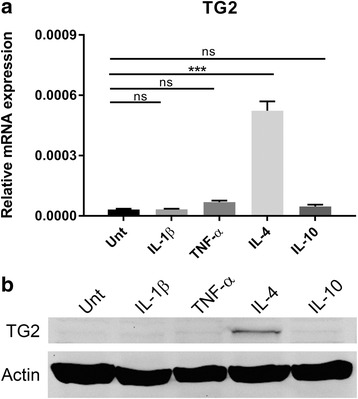
TG2 expression under different inflammatory stimuli. THP-1 monocytes were either untreated or treated with 50 ng/ml of IL-1β, TNF-α, IL-4, or IL-10 for 24 h and TG2 expression was analyzed by **a** qPCR or **b** WB. Data presented are the mean values + SEM (standard error of the mean) of at least three independent experiments. ***p < 0.001. Unt untreated, ns not significant

**Fig. 6:**
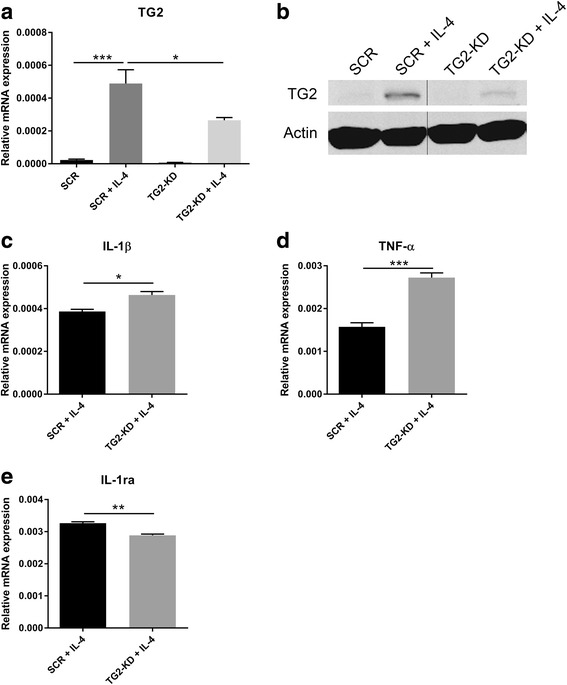
Knockdown of TG2 expression drives IL-4-activated THP-1 monocytes into a pro-inflammatory phenotype. Lentiviral particles were used to efficiently knockdown TG2 expression in THP-1 monocytes as confirmed both by **a** qPCR or **b** WB. To study the phenotype of TG2-KD cells, mRNA expression levels of **c** IL-1β, **d** TNF-α, and **e** IL-1ra were measured by qPCR in SCR or TG2-KD THP-1 monocytes activated for 24 h with IL-4 (50 ng/ml). Data presented are the mean values + SEM (standard error of the mean) of at least three independent experiments. *p < 0.05, **p < 0.01, ***p < 0.001. SCR scramble control, TG2-KD TG2 knockdown

To determine which inflammatory stimulus modulates TG2 expression in monocytes, as might occur during MS, THP-1 monocytes were either untreated or treated with two pro-inflammatory stimuli, i.e., IL-1β or TNF-α or with two anti-inflammatory stimuli, i.e., IL-4 or IL-10. The qPCR analysis revealed that TG2 mRNA levels were highly upregulated by IL-4 treatment (Fig. 5a). WB analysis confirmed the upregulation of TG2 protein in IL-4-treated monocytes (Fig. 5b). Subsequently, we studied the relevance of TG2 in determining the phenotype of IL-4-treated monocytes, by using TG2-KD or SCR THP-1 cells. First, we showed that in TG2-KD cells, TG2 mRNA (Fig. 6a) and protein levels (Fig. 6b) were significantly decreased after IL-4 treatment compared to IL-4-treated SCR cells. In addition, IL-4-treated TG2-KD cells expressed significantly more of the pro-inflammatory cytokines IL-1β and TNFα (Fig. 6c, d, respectively). Moreover, we observed a significant reduction in the mRNA level of the anti-inflammatory cytokine IL-1ra (Fig. 6e) in IL-4-treated TG2-KD monocytes.

### Effect of TG2-KD on adhesion and on cytoskeleton rearrangement in IL-4-treated THP-1 cells

**Fig. 7:**
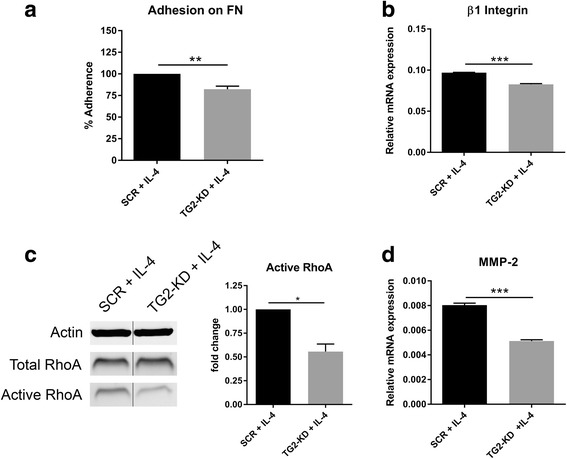
Effect of TG2 knockdown on the adhesion/migration and cytoskeleton rearrangement in IL-4-activated THP-1 monocytes. SCR or TG2-KD monocytes were treated with IL-4 (50 ng/ml) for 24 h and then examined for **a** their ability to adhere onto a FN layer for 1 h, **b** mRNA expression of β1 integrin, **c** activation of the small GTPase protein RhoA, and **d** mRNA expression of MMP-2. Data presented are the mean values + SEM (standard error of the mean) of at least three independent experiments. *p < 0.05, **p < 0.01, ***p < 0.001. SCR scramble control, TG2-KD TG2 knockdown

To identify relevant functional aspects of TG2 in IL-4-treated THP-1 monocytes, we studied adhesion properties. We observed that knockdown of TG2 expression resulted in less cell adhesion onto FN in response to IL-4 (Fig. 7a). As β integrins can act in concert with TG2 to promote cell adhesion, it was of interest to observe that IL-4-treated TG2-KD cells expressed significantly less β1 integrin (Fig. 7b). In addition, we observed a reduction of almost 50% of active RhoA GTPase, indicative of reduced cytoskeletal rearrangement, in IL-4-treated TG2-KD monocytes compared to SCR monocytes (Fig. 7c). Furthermore, IL-4-treated TG2-KD monocytes expressed significantly less MMP-2 (Fig. 7d), a protein known to be involved in the cleavage of ECM proteins and therefore permissive for cell migration [30, 31].

## Discussion

Alteration in TG2 expression and/or cross-linking activity has been extensively implicated in the pathogenesis of various human diseases, including neurodegenerative disorders [32], coeliac disease [33], and cancer [34]. Thus, based on our previous observations in rat and mouse models for MS, we here questioned whether monocyte-derived TG2 expression was altered in MS patients. Although a limited number of patients was included in the present study, we are the first to show that TG2 mRNA levels are significantly enhanced in monocytes derived from MS patients compared to HC subjects. The presence of low levels of TG2 in human monocytes is known for many years. Moreover, inflammatory factors have been shown to enhance TG2 levels in these cells [35–38]. Thus far, few studies have been performed to determine a physiological or pathological expression of TG2 in monocytes/macrophages in vivo. One of which is in a mouse model of atherosclerosis, showing increased TG2 immunoreactivity in infiltrated macrophages in the lesions, limiting lesion size [39].

As inflammation can alter TG2 expression, we characterized the inflammatory status of MS patient-derived monocytes and revealed that these cells expressed significantly lower mRNA levels of IL-1β. Interestingly, expression levels of either TNF-α or anti-inflammatory IL-1ra and TGF-β1 were unaffected.

Although, we cannot exclude the presence of separate cell populations with each specific pro- or anti-inflammatory phenotype, our data suggest that MS patient-derived monocytes have a mixed inflammatory phenotype, which is in agreement with the intermediate activation status displayed by monocyte-derived macrophages in active demyelinating MS lesions [40].

Noteworthy, elevated β3 integrin mRNA levels in MS patients indicate an increase migratory capacity of the cells [41, 42]. On the contrary, mRNA expression levels of the adhesion molecule β1 integrin were not affected.

In addition, our observation that TG2 does not correlate either with IL-1β or TNF-α expression but correlated positively with the expression of two anti-inflammatory markers, i.e., IL-1ra and TGF-β1 in MS monocytes, suggests that MS patient-derived monocytes expressing higher levels of TG2 have a more anti-inflammatory profile. We thus questioned what factor(s) could enhance monocyte-derived TG2, whose expression is known to be modulated by several inflammatory stimuli [27, 43, 44], due to the presence of various inflammatory factor-related response elements in the promotor region [45]. As inflammation occurs during MS pathogenesis, we determined the effect of inflammatory mediators on TG2 expression. In THP-1 cell, known to be responsive to inflammatory stimuli [46, 47], predominantly IL-4 induced TG2 mRNA and protein upregulation. These data suggest that the increased TG2 mRNA expression levels observed in MS patient-derived monocytes could, directly or indirectly, be mediated by circulating or autocrine IL-4, whose presence was described to be enhanced both in serum and in mononuclear cells of MS patients compared to HC subjects [48–51]. Subsequently, we found that knockdown of TG2 resulted in a pro-inflammatory phenotype of IL-4-treated THP-1 monocytes as indicated by the increased mRNA levels of IL-1β and TNF-α and by the reduced mRNA levels of the anti-inflammatory cytokine IL-1ra. These data suggest that the presence of TG2 drives an anti-inflammatory phenotype of monocytes. In agreement with this observation, TG2 was recently established as an anti-inflammatory marker of IL-4-treated human and mouse macrophages [52].

Furthermore, it is known that IL-4 affects adhesive/migratory properties of several cell types [53, 54]. Our findings regarding the attenuated adhesion, cytoskeleton rearrangement, and MMP-2 expression of IL-4-treated TG2-KD monocytes are the first to point out that these IL-4-mediated processes in human monocytes are, at least partly, regulated through TG2.

## Conclusion

In this study, we established that TG2 mRNA levels are increased in monocytes derived from MS patients and correlates with anti-inflammatory cytokine expression, proposing a more anti-inflammatory status of the TG2-expressing monocytes in MS. Furthermore, IL-4 is an important regulator of TG2 expression in THP1 monocytes and TG2 mediates IL-4-induced anti-inflammatory status of these cells as well as adhesion and cytoskeletal rearrangement in vitro. We thus propose that IL-4 upregulates TG2 expression in monocytes of MS patients, driving them into an anti-inflammatory status. This leads to the speculation that TG2 can mediate the enhanced adhesion of anti-inflammatory-tuned monocytes to the CNS endothelium of MS patients.

## Additional file

Additional file 1: TG2 expression in untreated and drug-treated MS patients. qPCR analysis was performed to detect TG2 in primary human monocytes isolated from untreated (*N* = 10) and drug-treated (*N* = 5) MS patients. Data are shown in box-and-whisker plots in which the median is represented by the horizontal line within the box, and the lower and upper whiskers represent the 5 and 95 percentiles. (TIFF 1769 kb)

## Abbreviations

ANOVA: One-way analysis of variance
BBB: Blood-brain barrier
BCA: Bicinchoninic acid
CNS: Central nervous system
DMT: Disease-modifying therapies
DTT: Dithiothreitol
EAE: Experimental autoimmune encephalomyelitis
ECM: Extracellular matrix
FN: Fibronectin
GAPDH: Glyceraldehyde-3-phosphate-dehydrogenase
HC: Healthy control
IL: Interleukin
IL-1ra: IL-1 receptor antagonist
MMP: Matrix metalloproteinase
MS: Multiple sclerosis
NGF: Nerve growth factor
PBMC: Peripheral blood mononuclear cells
PBS: Phosphate-buffered saline
PMSF: Phenylmethylsulfonyl fluoride
POLR2F: Polymerase II polypeptide F
PP-MS: Primary progressive
qPCR: Semi-quantitative real-time PCR
RR-MS: Relapsing-remitting MS
RT: Room temperature
SCR: Scramble control
SD: Standard deviation
SDS: Sodium dodecyl sulfate
SEM: Standard error of the mean
shRNA: Short hairpin RNA
TG2: Tissue transglutaminase or transglutaminase 2
TG2-KD: TG2-knockdown
TGF: Transforming growth factor
TNF: Tumor necrosis factor
Unt: Untreated
WB: Western blotting
