# Supplementary figures and images for: Monocyte-derived tissue transglutaminase in multiple sclerosis patients: reflecting an anti-inflammatory status and function of the cells?
Source: J Neuroinflammation. 2017 Dec 21;14:257. doi: 10.1186/s12974-017-1035-y (PMC5740592; doi:10.1186/s12974-017-1035-y)

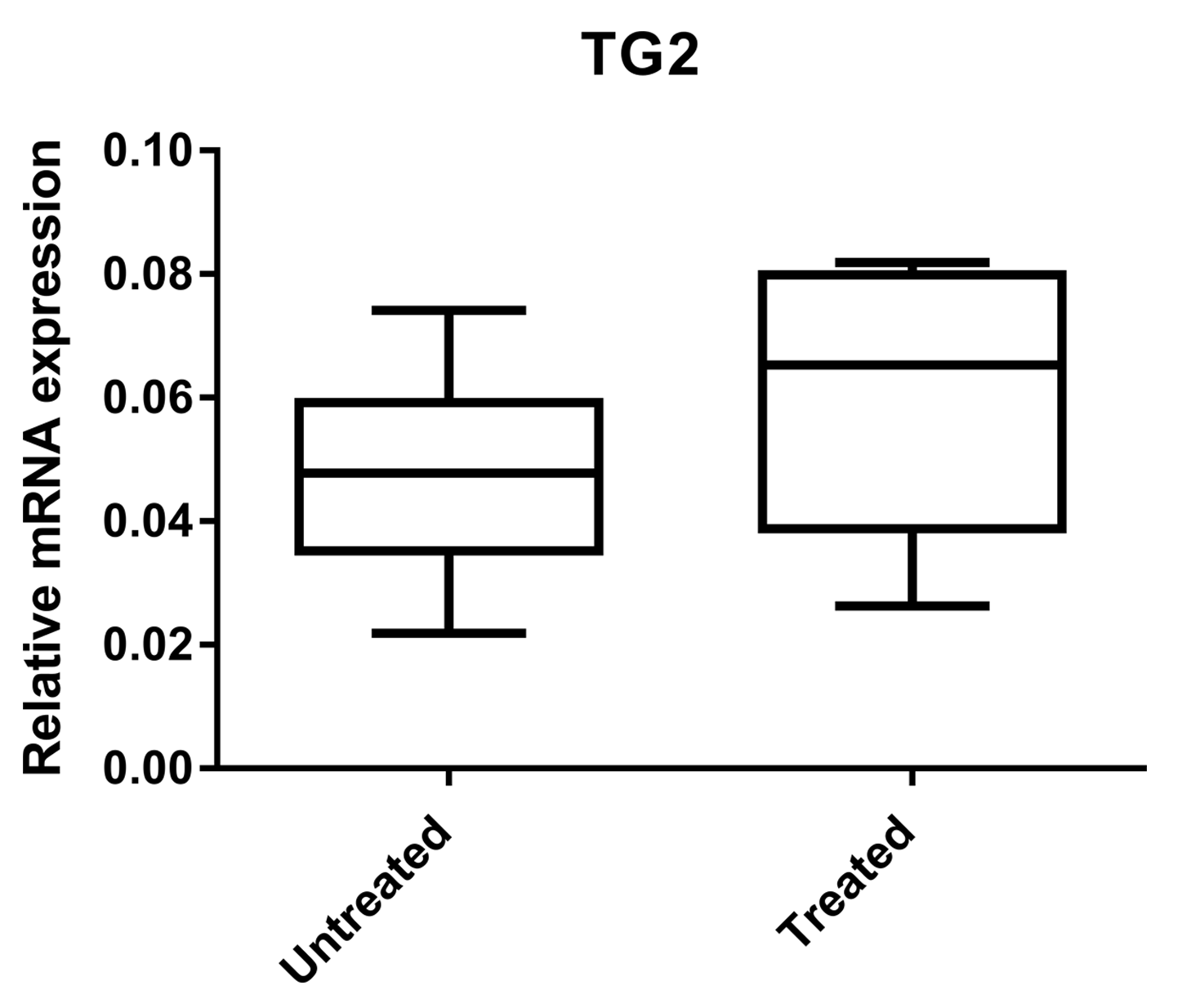

Supplement: Additional file 1: — TG2 expression in untreated and drug-treated MS patients. qPCR analysis was performed to detect TG2 in primary human monocytes isolated from untreated (N = 10) and drug-treated (N = 5) MS patients. Data are shown in box-and-whisker plots in which the median is represented by the horizontal line within the box, and the lower and upper whiskers represent the 5 and 95 percentiles. (TIFF 1769 kb) [file 12974_2017_1035_MOESM1_ESM.tif]
